# Thermal necrosis-aided dental implant removal: A rabbit model pilot study

**DOI:** 10.4317/medoral.25616

**Published:** 2023-02-18

**Authors:** Güzin Neda Hasanoğlu-Erbaşar, Mustafa Güngörmüş, Ebru Alimoğullari, Sevil Çayli, Elif Peker, Abdulkadir Narin, Metin Orhan

**Affiliations:** 1 Department of Oral and Maxillofacial Surgery, Faculty of Dentistry, Ankara Yıldırım Beyazıt University, Ankara, Turkey; 2 Department of Biomedical Engineering, Faculty of Engineering and Natural Sciences, Ankara Yildirim Beyazit University, Ankara, Turkey; 3 Department of Basic Sciences, Faculty of Dentistry, Ankara Yildirim Beyazit University, Ankara, Turkey; 4 Department of Histology and Embryology, Faculty of Medicine, Ankara Yıldırım Beyazıt University, Ankara, Turkey; 5 Department of Oral and Maxillofacial Surgery, Faculty of Dentistry, Gazi University, Ankara, Turkey; 6 Private Practice, İstanbul, Turkey; 7 Department of Orthodontics, Faculty of Dentistry, Ankara Yıldırım Beyazıt University, Ankara, Turkey

## Abstract

**Background:**

The significant advances in the materials and biological aspects of dental implants haven’t completely eradicated the implant failures. The removal of osseointegrated but otherwise failed implants present several challenges including adjacent tissues damage and necessity of bone augmentation for reimplantation. Controlled thermal necrosis has emerged as an alternative technique to aid removal of osseointegrated dental implants with minimal to no defect to healthy bone or surrounding tissues. This study aimed to evaluate the thermal necrosis-aided implant removal method in a rabbit osseointegration model.

**Material and Methods:**

A total of 8 male New Zealand rabbits were used in the study. Two dental implants were placed on each femur of the rabbits. Heating of the implants was performed after 7 weeks following the implantation. Heating was done by contacting the tip of an electrosurgey tool in monopolar mode at different power settings and contact durations (5W – 2 seconds, 5W – 10 seconds, and 10 W – 10 seconds). No heating was done on the control group. Implant stability right after implantation, before heat application and after heat application was determined using an Osstell™ Mentor Device. Following the removal of implants histological analyses were performed to determine the effects of heat application at cellular level.

**Results:**

ISQ values of the 10W-10s group was significantly lower compared to the other groups (*p*<0.001). No indication of progressive necrosis or irreversible damage was observed in any of the groups. However, the percent of empty-apoptotic lacunae were statistically higher in the 5W-10s and the 10W-10s groups compared the control and the 5W-2s groups.

**Conclusions:**

Within the conditions of this study, we conclude that heat application with an electrosurgery tool using monopolar mode at 10W power for 10 seconds is optimal for reversing osseointegration with no extensive or progressive damage to the bone.

** Key words:**Animal model, dental implant, implant removal, thermal necrosis.

## Introduction

Despite the significant advances in the materials and biological aspects of dental implants, implant failure still presents as an unavoidable risk. Implant failures are usually categorized as primary failure and secondary failure based on the underlying reason (1). Primary failure involves the implants that fail to osseointegrate. Factors related to improper implant design, improper surgical procedures, or patients’ status of health can lead to primary failure (2-5). In a retrospective cohort study conducted on 194 patients who presented dental implant failure during a 6-year period, lack of osseointegration has been found to constitute 38.1% of the failures (6). Other factors leading to implant failure and failure rates have been listed as peri-implantitis with 19.1%, overload with 24.7%, implant brake with 3.1% lack of augmentation with 1% and unknown reasons with 13.9% (6).

When a biological complication develops in the tissues surrounding an osseointegrated implant, a mechanical debridement and antibiotic and/or antiseptic treatment can be applied. However, if these treatments fail to resolve the complication, the surgeons may need to resort to removal of the implant. With a progressed bone loss around the implant and/or failed osseointegration, removing the implant with applying a reverse torque is a viable option. However, with osseointegrated but otherwise failed implants, reverse torque technique present several challenges (7). Removal of an osseointegrated implant may require cutting a 0.50-1.00 mm healthy bone tissue around the implant to weaken the bone-implant interface (8,9). In addition to need for removal of healthy bone tissue, there is also a risk of damaging surrounding tissues such as nerves, maxillary sinuses, neighboring teeth, etc. (7,10). Moreover, a 9-12 moths healing period and, depending on the size of the defect, bone augmentation may be necessary for reimplantation (10).

Controlled thermal necrosis has emerged as an alternative technique to aid removal of osseointegrated dental implants with minimal to no defect to healthy bone or surrounding tissues. The basic premise of this technique is heating the implant via electrosurgery tools or laser to increase the temperature of the implant and achieve a limited, non-progressive thermal necrosis at the bone-implant interface. The aim is to weaken the bone-implant interface and allow removal of implant via reverse torque with minimal to no damage to surrounding tissues. In early histomorphometry studies, it has been shown that at 47°C limited and non-progressive thermal necrosis occur at bone tissue. At 56°C, alkaline phosphatase is denatured and at 60°C extensive and potentially progressive thermal necrosis occurs (11,12). Therefore, proper techniques must be developed to prevent a temperature increase exceeding 47°C (12). Gungormus and Hasanoglu Erbasar have attempted to calculate the effect of different parameters, such as implant size, contact area and power on the duration required to reach 47°C at the bone-implant interface, using finite element analysis. They have concluded that at low power (5W) and 2.50 mm contact area, more than 4 seconds heat application was required to reach 47°C at the bone-implant interface, while the surface of the implant itself reached to this temperature at much shorter times (13). Kniha *et al*. have reported an *in vitro* study where the temperature thresholds were evaluated to assess the potential of thermal necrosis for implant removal. They have concluded that 51°C for 10 seconds and 5°C for 30 seconds resulted in significant bone matrix degeneration around the implant (14). Kawamura *et al*. have conducted an *in vivo* study on rats for implant removal using a high-frequency electrosurgical device. They have reported that all the implants were fractured during removal in the control group, while in the experimental group, the implants were removed without fracture. The bone necrosis was found to be localized within 50 µm of the bone-implant interface (15).

In this study, we have used a rabbit osseointegration model to evaluate the thermal necrosis-aided implant removal. We have used resonance frequency analysis (RFA) to monitor the time-wise changes in implant stability and measured the removal torques. Lastly, we have performed histological analyses to determine the effects of heat application at cellular level.

## Material and Methods

- Ethical Statement

The study was approved by the local ethical committee of the Kobay Experimental Animals Inc. (Ankara, Turkey), where the study was carried out (Approval date: 15.09.2017, File number: 243). The study was designed prospectively and carried out in accordance with the “Regulation on the Welfare and Protection of Animals Used for Experimental and Other Scientific Purposes”, published in the Official Gazette of the Republic of Turkey, No. 28141. The study was reported following the Animal Research: Reporting of In Vivo Experiments (ARRIVE) guidelines.

- Study Design and Sample Size

**Figure 1:**
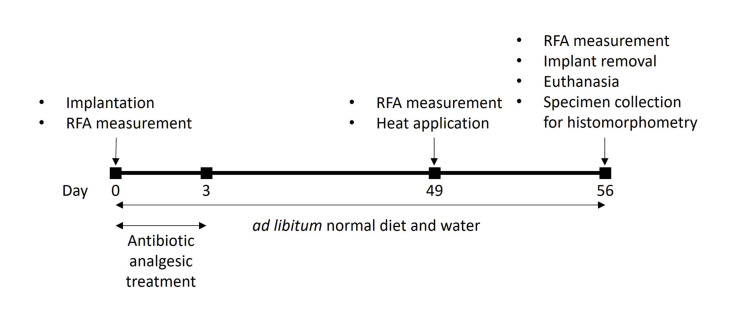
Schematic experimental timeline of rabbit osseointegration model, heat application and euthanasia.

The study was designed to determine the relationship between the implant stability, osteonecrosis and the power and duration of heat applied to the implants. A rabbit femur osseointegration model was used in the study. Male New Zealand rabbits (Oryctolagus cuniculus L) were used for the study. A power analysis with α=0.05, 95% confidence and 30% effect size determined a total sample size of *n*=24 with *n*=6 per group. Considering the animals or samples that may need to be excluded from the study due to death or other complications, a sample size of *n*=8 per group was used for 4 groups. Since 4 implants can be placed on each rabbit (left or right leg, proximal or distal site), a total of 8 animals were used in the study. The animals were caged individually. Each implantation site was considered as an experimental unit. A timeline diagram summarizing the study design is shown in Fig. 1.

- Exclusion criteria

Exclusion criteria were set as a weight loss exceeding 15% of the initial body weight within 2 weeks of implantation, local infection as indicated with severe lameness, persistent swelling, and discharge. Bone fracture during or post-implantation were additional exclusion criteria.

- Randomization

The implantation sites were not randomized but planned beforehand to ensure that all the experimental conditions were distributed among all the possible implantation sites (i.e., left leg – right leg, proximal site – distal site) to minimize any potential bias caused by the implantation site or an animal. (See supplementary information)

- Blinding

The surgeon who performed the implantation, heat application and RFA measurements was aware of the group allocations. The researcher who prepared the histological sections and the two evaluators who performed the evaluated the histomorphometry analyses were blinded to the group allocations.

- Outcome Measures

The outcome measures assessed were the implant stability quotient (ISQ) obtained from the RFA measurements, the implant removal torque, and the ratio of empty-apoptotic lacunae. ISQ was used to determine the sample size.

- Statistical Methods

Data control and statistical analyses were done using IMB SPSS Statistics Version 25 (IBM SPSS, Chicago, IL).

ISQ values at different time points were evaluated using one-way analysis of variance (ANOVA) test. The removal torque values were evaluated using Kruskal-Wallis H test. Interrater reliability of the lacunae counts by two evaluators were analyzed by intraclass correlation coefficient (ICC) analysis using two-way mixed effects, absolute agreement, multiple raters/measurements model. Occurrence of empty lacunae between groups was evaluated using cross tabulation (Chi-square test).

**Figure 2:**
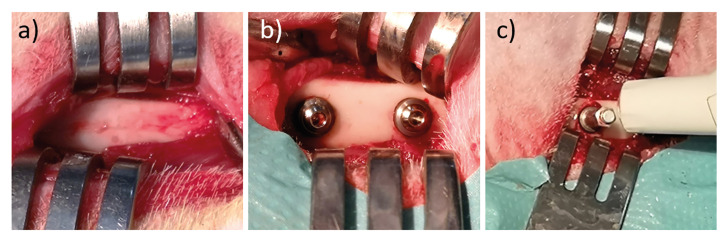
Digital photography images of a) blunt dissection, b) implants with the closing screws, and c) RFA measurement.

- Implantation

The animals were acclimatized in separate cages at 24±4°C, 50-65% humidity, and regular 12 hours daylight cycle for one week. All animals were provided food and water *ad libitum* throughout the study. An on-site veterinarian monitored and ensured the systemic health of rabbits. Prior to the operation, general anesthesia was achieved with intramuscular injection of 40 mg/kg Ketamin HCl (Alfamine, Ege-vet, İzmir, Turkey) and 5 mg/kg Xylazine (Alfazine, Ege-vet, İzmir, Turkey). Proximal regions of the femurs on the leg were shaved and 4% Articaine HCl + 1:100,000 epinephrine (Maxicaine, VEM, İstanbul, Turkey) local anesthetic injection was made. 3-4 cm long skin incisions were made towards the medial of the proximal metaphysis. The bones were exposed via blunt dissection. Implant sockets were prepared using 2 mm, 3.20 mm, and 3.70 mm implant burs successively. Lastly, the implant sockets were made ready using a 4 mm grooving bur. 10 mm space were left between each implant socket on the same bone. The implantation was made by applying clockwise torque using a calibrated torque wrench. Two OsseoSpeed TX Aqua 4.0S implants (Astra Tech Ltd., Gloucestershire, UK) were placed on each femur of the rabbits. After the first implant stability measurements, closing screws were placed on the implants (Fig. 2) and the incision lines were sutured. Post-operative analgesia and antibiotic treatment was maintained for 3 days with 1 mg/kg Tramadol HCl (Contramal, Abdi İbrahim İlaç San. ve Tic. A.Ş., İstanbul, Turkey) and 50 mg/kg Cefazolin (Sefazol, Mustafa Nevzat İlaç San. A.Ş., İstanbul, Turkey) injections, respectively. An on-site veterinarian monitored the animals for weight loss, lameness, infections, behavioral changes, and general health status.

- RFA

Implant stability right after implantation, before heat application and after heat application was determined using an Osstell ISQ device and Type 38 smartpegs (Osstell AB, Gothenburg, Sweden). The closing screws were removed and the smartpeg was placed on the implant. The handheld probe was positioned perpendicular to the longitudinal axis of the implants and the measurements were performed in four directions representing mesial, distal, lingual, and buccal. Measurements were repeated until the same implant stability quotient (ISQ) value was obtained three times consecutively for each direction. The average of the ISQ values obtained from four directions was considered as the ISQ of that implant.

- Heating of the implants

Heating of the implants was performed after 7 weeks following the implantation, under anesthesia as described before. ISQ values before heat application were measured and recorded. Heating was done by contacting the tip of an electrosurgey tool (EK-160, Üzümcü, Ankara, Turkey) in monopolar mode at different power settings and contact durations (5W – 2 seconds, 5W – 10 seconds, and 10 W – 10 seconds). No heating was done on the control group.

- Removal of the implants

ISQ values before implant removal were measured as described above. The implants were removed after 1 week following the heating under anesthesia. Counterclockwise torque was applied on the implants using a torque wrench. The highest torque value before the implant started to rotate within the socket was recorded as the removal torque.

- Histological examination

After the implants were removed, the animals were euthanized via intracardiac 22.5% KCl and 20 mg suxamethonium chloride. The regions on the femurs containing the implant sites were excised. The specimens were fixed in 10% neutral buffered formalin for 16 hours at 4°C and decalcified by immersion in formic acid for 8 weeks at 4°C. Then the specimens were dehydrated in ethanol series, cleared in xylene, and embedded in paraffin. 4 μm-thick serial sections were prepared transversely using a RM 2245 rotary microtome (Leica, Wetzlar, Germany). The sections were stained with hematoxylin–eosin and imaged using a BX43 light microscope (Olympus Corp., Tokyo, Japan) equipped with a DP26 digital imaging system (Olympus Corp., Tokyo, Japan). The fraction of empty lacunae and lacunae with visible apoptotic bodies were used to assess the degree of osteonecrosis among the groups. At least 200 lacunae from multiple images were counted within 0.50 mm distance from the implant-bone interface for each group by two evaluators independently using the multi-point tool of the ImageJ software (NIH, Bethesda, MD, USA).

- Statistical analysis

Data control and statistical analyses were done using IMB SPSS Statistics Version 25 (IBM SPSS, Chicago, IL). ISQ values at different time points were evaluated using one-way analysis of variance (ANOVA) test. The removal torque values were evaluated using Kruskal-Wallis H test. Interrater reliability of the lacunae counts by two evaluators were analyzed by intraclass correlation coefficient (ICC) analysis using two-way mixed effects, absolute agreement, multiple raters/measurements model. Occurrence of empty lacunae between groups was evaluated using cross tabulation (Chi-square test).

## Results

- Animal model

The study has begun with *n*=8 implants in each group. A total of 9 implants were excluded from the study due to bone fractures during implantation or post-surgery. To eliminate the potential effect of fracture healing on the osseointegration of the implants, these implants were not included in the final analysis. The resulting sample size was *n*=5 for the control group and *n*=6 for the remaining groups. No animal was lost during the study and the health statuses of the animals were within normal parameters.

- RFA and Removal Torque Measurements

**Figure 3:**
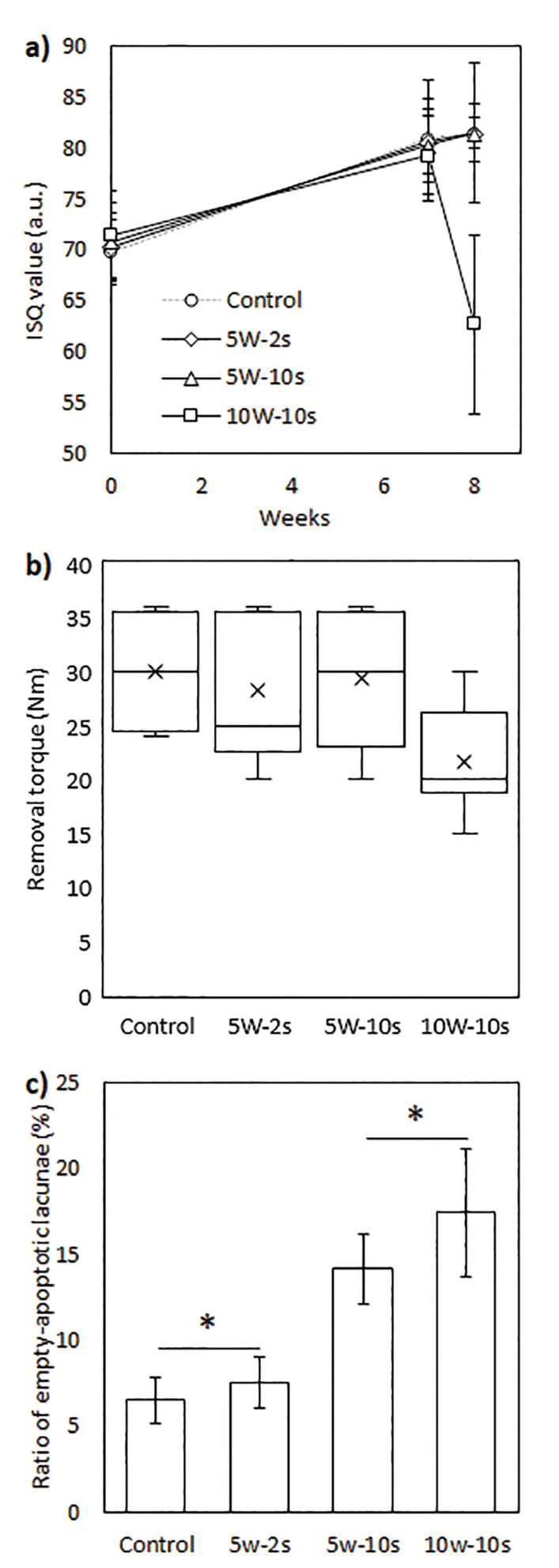
a) Timewise ISQ values of the implants. b) Removal torques of the implants 1 week after heat application. c) Ratio of the empty lacunae and lacunae with apoptotic bodies to normal lacunae (* indicates no statistically significant difference).

One-way ANOVA test showed no statistically significant differences in the ISQ values at the implantation time (F(3,19)=0.796, *p*=0.511) or before heat application (7 weeks after implantation) (F(3,19)=0.125, *p*=0.944). A statistically significant difference was observed after heat application (F(3,19)=23.262, *p*<0.001). ISQ values of the 10W-10s group was significantly lower (62.66 ± 8.80) compared to the other groups. No statistically differences were observed after heat application among the control (84.47 ± 6.83), 5W-2s (83.44 ± 2.83) and 5W-5s (85.44 ± 1.51) groups. (Fig. 3) Kruskal Wallis H test showed no significant differences in the removal torques (H(3)=5.610, *p*= 0.132). However, an empirical decrease in removal torque was observed in the 10W-10s group. (Fig. 3)

- Histological Analyses

**Figure 4:**
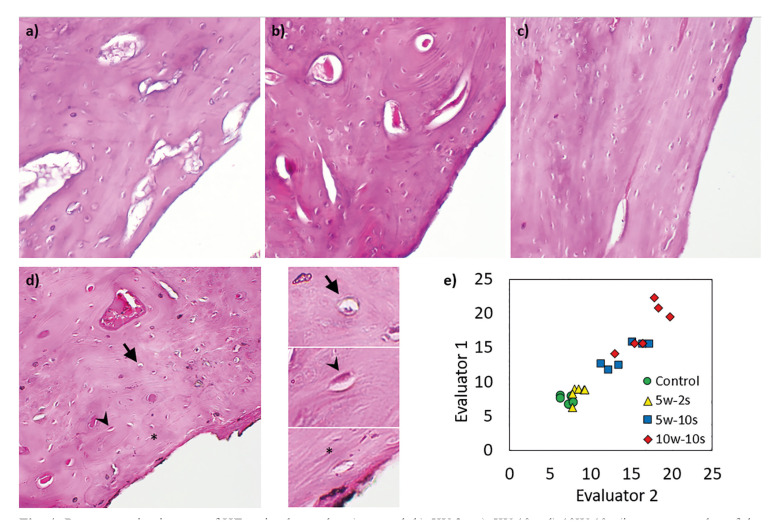
Representative images of HE stained samples a) control, b) 5W-2s, c) 5W-10s, d) 10W-10s (insets: examples of the characteristics evaluated; lacuna with apoptotic bodies (arrow), normal lacuna (arrowhead), empty lacuna (asterix)). e) Comparison of the necrosis evaluation by the two independent evaluators (each datapoint corresponds to results obtained from the same implant).

No indication of progressive necrosis or irreversible damage was observed in any of the groups. The ratio of empty lacunae and lacunae with apoptotic bodies to the normal lacunae was assessed by two evaluators independently. ICC test showed an excellent degree of reliability between the measurements of 23 individual specimens. The average measure ICC was 0.986 with a 95% confidence interval from 0.968 to 0.994 (F(22,22)= 69.548, *p* < 0.001). A cross-tabulation chi-square test was performed to examine the relation between the power output/time and the number of empty/apoptotic lacunae. The relation between these variables was found to be significant (X2 (3, N = 800) = 35.603, *p* < 0.001) The control and the 5W-2s groups, and the 5W-10s and the 10W-10s groups were found to be statistically similar. The percent of empty-apoptotic lacunae for the 5W-10s and the 10W-10s groups were statistically higher compared the control and the 5W-2s groups. (Fig. 3) Representative light microscopy images of the investigated samples and the correlation of the two independent evaluators are presented in Fig. 4.

## Discussion

Three different techniques (ISQ measurement, removal torque measurement, histological analysis) were used to evaluate the thermal necrosis aided implant removal in a rabbit implant model. A mixed trend was observed between the different techniques. While a clear statistically significant decrease was observed in the ISQ values for 10W-10s group after heat application, the decrease in the removal torque was empirical. Removal torque value is used as an indicator of the degree of osseointegration, higher removal torque being interpreted as a higher degree of osseointegration. This method has been used widely in both animal and clinical studies. However recent studies have drawn attention to the limitations of this method. In a computational study, Rittel *et al*. have shown that removal torque measurements cannot discriminate the degree of osseointegration beyond a relatively low level of 20% osseointegration (16). Multiple factors, such as bone-implant contact percentage, and the mechanical properties of the implant, effect the removal toque (17-19). In an earlier animal study done with implants with different surface properties, Koh *et al*. have suggested that removal torque measurement might not be the best test for evaluating osseointegration or the amount of bone around the implant (20). RFA, on the other hand, is a more direct measurement of the implant stability with reproducible results and independent of the implant type used (21-24).

We have based the power and duration of heat application based on our previous FEA study, where we have tested 5W and 40W power on three different implants, including the one used in this study (13). Our calculations suggested that at 5W power, the implant, and the bone would reach to 47°C at 2.5 and >4 seconds, respectively. We have tested this in the 5W-2s and 5W-10s groups. However, at 40W, the calculations showed the bone would reach to 47°C at ~2s. Therefore, 40W power was not used in this study due to the risk of overheating. A 10W-2s group was not included, since, according to calculations in the previous study, would not be enough to heat the bone to 47°C. Instead, 10W-10s was tested. Based on the calculations, we expected to observe the desired effect at 5W-10s, however, our findings revealed that this was not sufficient to reverse the osseointegration. The reversal of the osseointegration was achieved with 10W-10s.

Histological analyses based on the empty-apoptotic lacunae showed a similar degree of osteonecrosis between the 5W-10s and 10W-10s groups, which was higher compared to the control and the 5W-2s groups. Occasional empty lacunae are expected in healthy bone due to the shape of lacunae, positioning of nuclei, and the thickness of the sections (25). Therefore, presence of empty lacunae by itself is not regarded as an absolute indication of osteonecrosis. However, an increase in the number of empty lacunae and presence of some pyknotic nuclei (apoptotic bodies) is regarded as an indication of osteonecrosis (26). Histological findings for the 5W-10s group contradicted with the removal torque and RFA findings, which indicated no difference with the control and the 5W-2s groups. The contradicting mixed findings observed between the different techniques is likely due to the listed limitations and differences between the techniques.

The onset of progressive osteonecrosis in rabbits has been shown to occur as early as one week, as characterized with following thermally/mechanically or chemically induced osteonecrosis (27,28). In our study, the histological analyses were made 1 week after the heat application. Due to ethical concerns regarding the number of animals used in the study, study groups for histological analyses at multiple time points after heat application could not be included in the study. 1 week after heat application, no indication of progressive osteonecrosis or bone collapse was observed, which is usually characterized by complete loss of normal lacunae or severe inflammatory infiltrates in the intertrabecular spaces (29). Future studies, where the histological changes after heat application is investigated in a broader timeframe, may help elucidate the processes osteonecrosis and healing following heat application.

The findings from three different techniques indicate that heat application at 5W for 2 does not cause any changes in osseointegration or bone histology. Heat application at 5W for 10 seconds results in observable changes in bone histology, however, these changes do not reflect to the implant stability. Heat application at 10W for 10 seconds results in significant changes both in bone histology and implant stability.

One of the limitations of this study was the lack of different implant sizes. We have previously reported in a computational study that larger implants require longer heat application for the bone-implant interface to reach 47°C (13). Including different implant sizes would require a non-justifiable number of animals since it would still be difficult to draw generalizations based on implant size due to use of electrosurgery tools not intended for inducing thermal necrosis. Currently, there are several patent applications for devices designed for inducing thermal necrosis to aid implant removal (30). At the time of writing this manuscript, no such commercial or experimental devices were available. If such devices with standardized power settings and tip sizes become available, studies towards creating guidelines for thermal necrosis-aided implant removal will progress faster. In addition, all the studies on this topic to this date have been performed on healthy bone models. Since this is a relatively new technique, studies on compromised bone models, such as osteoporosis or peri-implantitis, are lacking.

## Conclusions

Within the conditions of this study, heat application with an electrosurgery tool using monopolar mode at 10W power for 10 seconds has been found optimal for reversing osseointegration with no extensive or progressive damage to the bone. Lower power setting and shorter duration had no effect on implant stability. Based on our findings and previous reports, using removal torque alone to assess thermal necrosis-aided implant removal is not suggested. Complementary methods, such as RFA, histological analyses, immunohistochemistry, etc. must be utilized. In summary, our findings contribute to the previous findings on the feasibility of thermal necrosis-aided implant removal and presents suggestions for further studies towards establishing the effectiveness of this method.

## References

[1] Chrcanovic B, Albrektsson T, Wennerberg A (2014). Reasons for failures of oral implants. J Oral Rehabil.

[2] Arys A, Philippart C, Dourov N, He Y, Le QT, Pireaux JJ (1998). Analysis of titanium dental implants after failure of osseointegration: Combined histological, electron microscopy, and X-ray photoelectron spectroscopy approach. J Biomed Mater Res.

[3] Koka S, Zarb G (2012). On Osseointegration: The Healing Adaptation Principle in the Context of Osseosufficiency, Osseoseparation, and Dental Implant Failure. Int J Prosthodont.

[4] Baires-Campos FE, Jimbo R, Bonfante EA, Fonseca-Oliveira MT, Moura C, Zanetta-Barbosa D (2015). Drilling dimension effects in early stages of osseointegration and implant stability in a canine model. Med Oral Patol Oral Cir.

[5] Castellanos-Cosano L, Rodriguez-Perez A, Spinato S, Wainwright M, Machuca-Portillo G, Serrera-Figallo MA (2019). Descriptive retrospective study analyzing relevant factors related to dental implant failure. Med Oral Patol Oral Cir.

[6] Manor Y, Oubaid S, Mardinger O, Chaushu G, Nissan J (2009). Characteristics of Early Versus Late Implant Failure: A Retrospective Study. J Oral Maxillofac Surg.

[7] Lee JB (2017). Selectable Implant Removal Methods due to Mechanical and Biological Failures. Case Rep Dent.

[8] Stajčić Z, Stajčić LS, Kalanović M, Đinić A, Divekar N, Rodić M (2016). Removal of dental implants: review of five different techniques. J Oral Maxillofac Surg.

[9] Levin L (2008). Dealing with dental implant failures. J Appl Oral Sci.

[10] Eriksson A, Albrektsson T (1983). Temperature threshold levels for heat-induced bone tissue injury: a vital-microscopic study in the rabbit. J Prosthet Dent.

[11] Eriksson RA, Albrektsson T, Magnusson B (1984). Assessment of bone viability after heat trauma. A histological, histochemical and vital microscopic study in the rabbit. Scand J Plast Reconstr Surg.

[12] Can M, Koluaçik S, Bahçe E, Gokce H, Tecellioglu FS (2022). Investigation of thermal damage in bone drilling: Hybrid processing method and pathological evaluation of existing methods. J Mech Behav Biomed Mater.

[13] Gungormus M, Erbasar GNH (2019). Transient Heat Transfer in Dental Implants for Thermal Necrosis-Aided Implant Removal: A 3D Finite Element Analysis. J Oral Implantol.

[14] Kniha K, Buhl EM, Hermanns-Sachweh B, Al-Sibai F, Bock A, Peters F (2021). Implant removal using thermal necrosis-an in vitro pilot study. Clin Oral Investig.

[15] Kawamura A, Akiba Y, Nagasawa M, Takashima M, Arai Y, Uoshima K (2021). Bone heating and implant removal using a high-frequency electrosurgical device: An in vivo experimental study. Clin Oral Implants Res.

[16] Rittel D, Dorogoy A, Shemtov-Yona K (2018). Modeling the effect of osseointegration on dental implant pullout and torque removal tests. Clin Implant Dent Relat Res.

[17] Stenlund P, Murase K, Stålhandske C, Lausmaa J, Palmquist A (2014). Understanding mechanisms and factors related to implant fixation; a model study of removal torque. J Mech Behav Biomed Mater.

[18] Elias CN, Oshida Y, Lima JHC, Muller CA (2008). Relationship between surface properties (roughness, wettability and morphology) of titanium and dental implant removal torque. J Mech Behav Biomed Mater.

[19] Chen S, Rittel D, Shemtov-Yona K (2022). Probing the sensitivity of the resonant frequency analysis to the dental implant-bone condition: A numerical study. J Mech Behav Biomed Mater.

[20] Koh JW, Yang JH, Han JS, Lee JB, Kim SH (2009). Biomechanical evaluation of dental implants with different surfaces: Removal torque and resonance frequency analysis in rabbits. J Adv Prosthodont.

[21] Degidi M, Daprile G, Piattelli A (2012). Primary stability determination by means of insertion torque and RFA in a sample of 4,135 implants. Clin Implant Dent Relat Res.

[22] Díaz-Castro MC, Falcao A, López-Jarana P, Falcao C, Ríos-Santos JV, Fernández-Palacín A (2019). Repeatability of the resonance frequency analysis values in implants with a new technology. Med Oral Patol Oral Cir Bucal.

[23] Herrero-Climent M, Santos-García R, Jaramillo-Santos R, Romero-Ruiz MM, Fernández-Palacin A, Lázaro-Calvo P (2013). Assessment of Osstell ISQ's reliability for implant stability measurement: A cross-sectional clinical study. Med Oral Patol Oral Cir Bucal.

[24] Guerrero-González M, Monticelli F, Saura García-Martín D, Herrero-Climent M, Ríos-Carrasco B, Ríos-Santos JV (2020). Reliability of the Resonance Frequency Analysis Values in New Prototype Transepithelial Abutments: A Prospective Clinical Study. Int J Environ Res Public Health.

[25] Öztürk K, Kış HC (2022). Peri-implant bone microstructural analysis and comparison of resonance frequency analysis before prosthetic placement: a retrospective study. Clin Oral Invest.

[26] Yamamoto T, Irisa T, Sugioka Y, Sueishi K (1997). Effects of pulse methylprednisolone on bone and marrow tissues. Corticosteroid-induced osteonecrosis in rabbits. Arthritis Rheum.

[27] Albrektsson T, Eriksson A (1985). Thermally induced bone necrosis in rabbits: relation to implant failure in humans. Clin Orthop Relat Res.

[28] Kabata T, Kubo T, Matsumoto T, Hirata T, Fujioka M, Takahashi KA (2005). Onset of steroid-induced osteonecrosis in rabbits and its relationship to hyperlipaemia and increased free fatty acids. Rheumatology.

[29] Fondi C, Franchi A (2007). Definition of bone necrosis by the pathologist. Clin Cases Miner Bone Metab.

[30] Winnen RG, Kniha K, Modabber A, Al-Sibai F, Braun A, Kneer R (2021). Reversal of Osseointegration as a Novel Perspective for the Removal of Failed Dental Implants: A Review of Five Patented Methods. Materials.

